# Genome Sequences of Nine *Bordetella holmesii* Strains Isolated in the United States

**DOI:** 10.1128/genomeA.00438-14

**Published:** 2014-06-19

**Authors:** Eric T. Harvill, Laura L. Goodfield, Yury Ivanov, William E. Smallridge, Jessica A. Meyer, Pamela K. Cassiday, Maria L. Tondella, Lauren Brinkac, Ravi Sanka, Maria Kim, Liliana Losada

**Affiliations:** a The Pennsylvania State University, University Park, Pennsylvania, USA; b Centers for Disease Control and Prevention, Atlanta, Georgia, USA; c J. Craig Venter Institute, Rockville, Maryland, USA

## Abstract

An increasing number of pertussis-like cases are attributed to the emergent pathogen *Bordetella holmesii*. The genomes of 9 clinical isolates show that they are clonal, lack the virulence factors encoded by *B. pertussis*, and are more similar to nonpertussis bordetellae. New markers for *B. holmesii* can be developed using these sequences.

## GENOME ANNOUNCEMENT

An increasing number of pertussis-like cases in the United States and Europe are attributed to the emergent pathogen *Bordetella holmesii* (1, 2). An analysis of the 2010-2011 outbreak in Ohio showed that *B. holmesii* was detected in nearly 20% of pertussis-like illnesses (1), up from ~1% in the 1990s (3, 4). Only three whole-genome sequences are available for this group of pathogens (5–7). *B. holmesii* genomes do not encode known *Bordetella pertussis* virulence factors, though they share a genomic island that contains the IS*481* insertion element (7) commonly used to diagnose *B. pertussis* infections and leading to common misidentifications. More specific diagnostic tests were developed using the *B. holmesii*-specific IS*1001* element (4), but it is unclear whether this marker is sufficient due to sparse genomic data.

Here, we report the genome sequences of 9 clinical isolates obtained between 2004 and 2011: six from patients with bacteremia (five from blood and one from synovial fluid) and three respiratory isolates from patients with pertussis-like symptoms. A respiratory isolate was from an infant who had a coinfection with *B. pertussis*. Genomic DNA was prepared (8) and sequenced using a combination of 3- or 5-kb mate-pair (~30× coverage) and 100-bp Illumina paired-end reads (~50× coverage). After quality trimming, all reads were used in assemblies with Celera Assembler 6.1 (9) or Velvet Assembler. Underlying consensus sequences and gaps were improved using custom scripts. All genomes had between 119 and 213 contigs (Table 1). The overall G+C content was ~62.6%, with genome sizes ranging from 3.55 Mb to 3.59 Mb.

**Table 1:** Isolate characteristics and accession numbers

*B. holmesii* isolate ID	State	Yr isolated	Source	Genome length (bp)	Total no. of contigs	GenBank accession no.
H572	Colorado	2010	Synovial fluid	3,585,459	119	JFZY00000000
H585	Minnesota	2010	Blood	3,587,402	150	JFZZ00000000
H629	New York	2010	Blood	3,475,248	190	JGVZ00000000
H635	California	2010	Respiratory fluid	3,569,022	173	JGAA00000000
H643	Pennsylvania	2010	Blood	3,614,976	193	JGWD00000000
H719	Minnesota	2011	Blood	3,578,998	149	JGWA00000000
H785	Oregon	2011	Respiratory fluid	3,565,090	161	JGWB00000000
H809	New York	2011	Blood	3,584,230	153	JMGZ00000000
04P3421	Massachusetts	2004	Respiratory fluid	3,595,240	213	JGWC00000000

*B. holmesii* isolates belonged to the same multilocus sequencing type (MLST), as seen in *B. pertussis* strains (10), suggesting that the *B. holmesii* isolates were also clonal. The genomes were annotated and predicted to have between 3,118 and 3,285 genes. The genomic content of the *B. holmesii* strains was more similar to those of *Bordetella avium* and *Bordetella petrii* than to those of *B. pertussis* or *B. bronchiseptica*. However, nearly 66% of the genes were shared with *B. pertussis* or *Bordetella bronchiseptica*. Almost 400 genes were shared by all *B. holmesii* isolates but were not present in any other bordetellae, likely due to acquisition via horizontal transfer. Many of these genes were involved in the transport and detoxification of organic compounds and antibiotics. Each strain had between 24 and 114 unique genes, including one strain that had residual members of a degraded type III secretion system, as seen in *Escherichia coli* (11). As expected, the IS*481* element was present in all genomes (32 to 65 copies), as was BhlIS*1001* (5 to 21 copies). The acellular vaccine targets of pertussis toxin, pertactin, and fimbriae were not present, while filamentous hemagglutinin was encoded by all *B. holmesii* genomes.

These findings suggest that circulating *B. holmesii* isolates in the United States emerged from a single genetic background more similar to nonpertussis bordetellae. The genomes are a resource for understanding the pathogenicity and evolution of *B. holmesii* and for further developing detection and differentiation strategies.

### Nucleotide sequence accession numbers.

The *B. holmesii* whole-genome shotgun project has been deposited at DDBJ/EMBL/GenBank under the accession no. described in Table 1. The version described in this paper is the first version.
